# Tehran Survey of Potential Risk Factors for Multiple Births

**DOI:** 10.22074/ijfs.2017.4700

**Published:** 2017-09-03

**Authors:** Reza Omani Samani, Amir Almasi-Hashiani, Samira Vesali, Fatemeh Shokri, Rezvaneh Cheraghi, Farahnaz Torkestani, Mahdi Sepidarkish

**Affiliations:** 1 Department of Epidemiology and Reproductive Health, Reproductive Epidemiology Research Center, Royan Institute for Reproductive Biomedicine, ACECR, Tehran, Iran; 2 Department of Obstetrics and Gynecology, Shahed University of Medical Sciences, Tehran, Iran

**Keywords:** Multiple Pregnancy, Pregnancy, Labor, Cross-Sectional Study, Prevalence Rate

## Abstract

**Background:**

The multiple pregnancy incidence is increasing worldwide. This increased incidence is concerning to the health care system. This study aims to determine the frequency of multiple pregnancy and identify factors that affect this frequency in Tehran, Iran.

**Materials and Methods:**

This cross-sectional study included 5170 mothers in labor between July 6-21, 2015 from 103 hospitals with Obstetrics and Gynecology Wards. The questionnaire used in this study consisted of five parts: demographic characteristics; information related to pregnancy; information related to the infant; information regarding the multiple pregnancy; and information associated with infertility. We recruited 103 trained midwives to collect data related to the questionnaire from eligible participants through an interview and medical records review. Frequencies and odds ratios (OR) for the association between multiple pregnancy and the selected characteristics (maternal age, economic status, history of multiple pregnancy in first-degree relatives, and reproductive history) were computed by multiple logistic regression. Stata software, version 13 (Stata Corp, College Station, TX, USA) was used for all statistical analyses.

**Results:**

Multiple pregnancy had a prevalence of 1.48% [95% confidence interval (CI): 1.19-1.85]. After controlling for confounding variables, we observed a significant association between frequency of multiple pregnancy and mother’s age (OR=1.04, 95% CI: 1.001-1.09, P=0.044), assisted reproductive technique (ART, OR=6.11, 95% CI: 1.7- 21.97, P=0.006), and history of multiple pregnancy in the mother’s family (OR=5.49, 95% CI: 3.55-9.93, P=0.001).

**Conclusion:**

The frequency of multiple pregnancy approximated results reported in previous studies in Iran. Based on the results, we observed significantly greater frequency of multiple pregnancy in older women, those with a history of ART, and a history of multiple pregnancy in the mother’s family compared to the other variables.

## Introduction

The occurrence of twin and multiple pregnancies has increased in developed countries (1) and is associated with concern in the health care system. Multiple pregnancy results in premature delivery, underweight newborns, and increased congenital anomalies. The worst outcome is maternal and neonatal mortality (2). The existing evidence shows a significantly lower one-year survival in multiple infants compared to singletons. The frequency of growth disorders, as well as physical and mental disabilities is higher in multiple newborns, if the infants survive (3, 4). A few studies conducted in Iran have reported a frequency of twin pregnancy from 1.5 to 8% (5-7). However, these studies were frequently conducted in one or several hospitals. Most were retrospective studies that reviewed the records. Inconsistency in reporting the frequency of multiple pregnancy could be due to structural differences in the populations studied and design effect and systematic errors (selection or information bias), in addition to changes in the frequency of the interested outcome over time (8). Hence, it is necessary to accurately identify the frequency of multiple pregnancy and impacting factors which lead to identification of high-risk groups and increased care for these groups, and assists authorities and policy makers in evidence-based decision making to increase cost effectiveness of the interventions. The aim of this study is to determine the frequency of twin and multiple pregnancy and to identify factors that affect the frequency of this phenomenon in Tehran Province, one of the main provinces in Iran.

## Materials and Methods

We conducted a cross-sectional study in Tehran Province, Iran which included the twenty-fifth most populated city worldwide-the capital of Iran (9). Participants comprised 5170 mothers in labor between July 6 to 21, 2015 who referred to the Obstetrics and Gynecology Wards of 103 hospitals. These hospitals are affiliated with Tehran, Beheshti, and Iran medical universities, which oversee and mange 19 (Tehran), 43 (Beheshti), and 41 (Iran) hospitals. We included all women in this study regardless of the type of delivery (natural or cesarean section) and the pregnancy outcome (live birth, stillbirth, or spontaneous abortion).

The Ethical Committee of Royan Institute approved this study (EC/92/1097). All participants received complete explanations about the study aims and data confidentiality, which mentioned their complete freedom to participate. Eligible individuals were also assured that acceptance or refusal to participate in the research had no influence on their treatment procedures. Completion of the questionnaire was considered as written informed consent.

According to the 2% prevalence of multiple pregnancy in the population (10), the effect size of 0.006 and a design effect of approximately 2, we estimated the required minimum sample size to be approximately 4181 pregnant women (α=0.05). The dependent variable studied was multiple pregnancy (twin or higher). The questionnaire used in the study included five parts: demographic characteristics (13 items); information related to pregnancy (26 items); information related to the infant (15 items); information regarding the multiple pregnancy (18 items); and information associated with infertility (7 items).

### Face and content validity

A total of 10 experts in gynecology, sexology, and methodology assessed face and content validity of the questionnaire. The validity index for each question and total validity were calculated (11). To equalize the experts’ perceptions of content validity indices (relevancy, clarity, and comprehensiveness of the tool), we sent the definitions of these indices with the questionnaire. Relevancy, clarity, and comprehensiveness were defined as follows. Relevancy was the ability of selected questions in order to reflect the content, lucidity of the questions concerned their wording, concept was clarity, and the instrument’s ability to include all content domains or areas was considered comprehensiveness. The experts were asked to review clarity and relevancy of each item, and comprehensiveness of the total questionnaire. Scores were given as: 1 (inappropriate), 2 (somewhat appropriate), 3 (appropriate), and 4 (quite appropriate). Experts’ responses were gathered within 1 to 3 weeks (12).

Data related to the questionnaire was collected from eligible participants through interviews conducted by 103 trained midwives. If pain was a barrier to mothers’ responses to the questionnaire, data were taken after childbirth at the time of admission in the hospital, which took 24 hours. To ensure valid and reliable data collection, the following actions were taken. We conducted three training sessions for midwives who collected the data. In these sessions, the correct way to collect data, definition of variables, and creation of a common perception among midwives were considered. A pilot study for operational feasibility and identification of implementation problems and difficulties related to the questionnaire was conducted in five hospitals. A visit to hospitals without previous coordination for examining how to complete the questionnaires.

### Statistical analysis

Categorical and continuous variables were summarized as number (%) and mean (SD). The frequency of multiple pregnancies was calculated as the percentage of multiple pregnancies by mother’s age, history of infertility, assisted reproductive technique (ART), history of multiple pregnancy in the mother’s family, history of multiple pregnancy in the father’s family, the mother born of multiple pregnancy, and the father born of multiple pregnancy. Crude odds ratios (OR) for the association between the selected characteristics (maternal age, economic status, a history of multiple pregnancy in first-degree relatives, and reproductive history) and multiple pregnancy were computed by univariate logistic regression. In the analysis we considered hospitals as a cluster. Multivariate logistic regression was used to adjust OR simultaneously for the aforementioned variables. Criteria for model building was based on the Hosmer-Lemeshow method (13). Results were presented as OR with 95 % confidence intervals (CI) (14). The Hosmer-Lemeshow test was used for goodness of fit of the model (15). We used Stata version 13 (Stata, College Station, TX, USA) for statistical analysis.

## Results

In this study, the IRA (Inter Rater Agreement) relevancy of the questions was 78.34% with a clarity of 92.78%. The questionnaire had a total relevancy of 86.23%, clarity of 87.48%, and comprehensiveness of 82%. In this survey we examined 5170 eligible pregnant women. Among the examined pregnancies, there were 5093 single cases and 77 multiple cases. Multiple pregnancy had a frequency of 1.48% (95% CI: 1.19-1.85). Mothers had a mean age of 29.23 years (95% CI: 29.08-29.38). Mothers with single pregnancy had a significantly lower mean (SD) age of 29.20 (5.46) compared to 30.98 (5.86) for mothers with multiple pregnancy (P=0.004, Table 1).

**Table 1:** Association between multiple pregnancy and potential predictors


Variable		Multiple pregnancies		Crude OR	95% CI for OR
		Yes	No				

Mother’s age (Y)					
	Mean (SD)	30.98 (5.86)	29.20 (5.46)	1.05	1.01-1.10
Type of pregnancy					
	Wanted	63 (1.56)	4069 (98.48)	1	-
	Unwanted	14 (1.37)	1006 (98.63)	0.89	0.50-1.61
History of infertility					
	No	55 (1.15)	4719 (98.85)	1	-
	Yes	22 (5.70)	364 (94.30)	5.18	3.12-8.59
Received assisted reproductive technique (ART)					
	No	57 (1.16)	4876 (98.84)	1	-
	Yes	20 (8.44)	217 (91.56)	7.88	4.65-13.35
History of multiple pregnancy in mother’s family					
	No	24 (0.62)	3832 (99.38)	1	-
	Yes	53 (4.05)	1257 (95.95)	6.73	4.13-10.94
History of multiple pregnancy in father’s family					
	No	50 (1.20)	4100 (98.80)	1	-
	Yes	27 (2.66)	989 (97.34)	2.23	1.39-3.59
Mother as the outcome of multiple pregnancy					
	No	74 (1.45)	5014 (98.55)	1	-
	Yes	3 (3.85)	75 (96.15)	2.71	0.83-8.79
Father as the outcome of multiple pregnancy					
	No	75 (1.47)	5012 (98.53)	1	-
	Yes	2 (2.53)	77 (97.47)	1.73	0.41-7.19


OR; Odds ratio and CI; Confidence interval.

There were 237 (4.58%) cases treated with ART. The frequency of multiple pregnancy was 1.15% in women who did not receive ART (95% CI: 0.08- 1.49), while the frequency of multiple pregnancy was 8.44% in women who received ART (95% CI: 5.5-12.72). Using logistic regression analysis, we estimated the OR for the association between ART and multiple pregnancy to be approximately 7.88 (95% CI: 4.65-13.35, P<0.001). Hence, the frequency of multiple pregnancy in women who received ART was 7.88 times greater.

As seen in Table 2, a significant association existed between variables such as mother’s age (OR=1.04, 95% CI: 1.001-1.09, P=0.044), ART (OR=6.11, 95% CI: 1.7-21.97, P=0.006), and history of multiple pregnancy in the mother’s family (OR=5.49, 95% CI: 3.55-9.93, P=0.001) with the frequency of multiple pregnancy after controlling for other variables in this table. No significant association existed between the frequency of multiple pregnancy and other variables. The goodness of fit test was performed for the final version, which showed a good fit of the model (Hosmer- Lemeshow chi2=5.57, P=0.695).

**Table 2:** Demographic characteristic and the first birth interval according to gender


Variable	Adjusted OR	95% CI	P value

Mother’s age (Y)	1.04	1.01-1.09	0.044
ART	6.11	1.70-21.97	0.006
History of multiple pregnancy in mother’s family	5.94	3.55-9.93	0.001
Type of pregnancy	0.81	0.44-1.51	0.518
History of infertility	0.94	0.27-3.25	0.929
History of multiple pregnancy in father’s family	1.31	0.79-2.17	0.293
Mother as the outcome of multiple pregnancy	1.30	0.38-4.47	0.669
Father as the outcome of multiple pregnancy	1.88	0.43-8.24	0.399


ART; Assisted reproductive technique, OR; Odds ratio, and CI; Confidence interval.

## Discussion

After remarkable reduction in multiple births during the second half of the twentieth century, most recently a steady increase exists in multiple births and its adverse subsequent consequences worldwide (16). Studies have shown that the majority of this increase is due to the increased age at pregnancy and the emergence of ART. In the United States from 1972 to 1999, there were 6 times more triplets and 12 times more multiples than the past. If women who became pregnant at an older age were considered in the calculation, the above prevalence would increase approximately 50-60 times (17). Iran, like other developing countries, has experienced major changes in the structure of its population. The socio-economic development and establishment of health care networks caused major changes in indicators of population health and epidemiology in Iran (18, 19). Demographic information, mainly derived from the census10 years once in Iran, along with health indicators confirmed the above mentioned. Fertility indicators (birth and total fertility rate) showed that since 2000, Iran has experienced a downward trend and the population growth rate has been close to one. By taking into account the age composition of the community, we have found that the population is increasing in age, whereas the relative frequency of marriage has decreased and the age of marriage increased in both men and women (9). During the last 10 years, no study has evaluated the frequency of multiple pregnancy and its trend. With regard to information obtained in a few studies, the results have suggested a subtle increase in multiple births in Iran. The highest frequency reported was 2% estimated from the last study conducted in 2005 in three large teaching hospitals in Tehran (10). In the current study, multiple pregnancy had a frequency of 1.48 (95% CI: 1.19-1.85), which approximated the frequency reported in previous studies. The rate has been affected by Genetics agents and ART. Therefore, it differs in various regions of the world. Bortolus et al. (20) carried out a systematic review on the epidemiology of multiple births. The results showed a higher frequency of multiple births in African countries and the black race compared with other countries. The lowest frequency was reported from Japan and Southeast Asian countries. Our results showed a moderate rate in Iran.

An international committee for monitoring ART suggested that one embryo should be transferred per cycle (21). Saraswat et al. (22) conducted a systematic review in 2010. The results indicated that infertility centers increased the number of embryos transferred (sometimes up to 4 embryos) according to domestic law and patient preference. In the current study, the OR for an association between ART and multiple pregnancy was estimated at 7.88 (95% CI: 4.65-13.35), which confirmed findings of other studies (23). Another systematic review on studies from 1950 to 2010 in the United States revealed that 20% of twins, 40% of triplets, and 71% of other types of multiple pregnancy were caused by ovarian stimulation whereas 16% of twins, 45% of triplets, and 30% of other types were the result of IVF (16).

Martikainen et al. (24) conducted a study in four centers in 2001. The results showed the clinical pregnancy rate per transfer was 32.4% in the one embryo transfer group and 47.1% in the two embryo transfer group. The relative risk for twin birth was 10.18. Mclernon et al. (25) reported a relative risk for twin birth of approximately 24.4 (95% CI: 3.42-173.8), which indicated a very high risk for twin pregnancy after the transfer of two or more embryos. A review study conducted in 2002 by De Sutter et al. (26) compared double embryo transfer (DET) to single embryo transfer (SET) according to the results of the 7407 cycles in 6 cohort studies. Overall, the pregnancy success rate had no significant difference between the two procedures (SET: 33.9%-DET: 35%) Twins were 1% in SET which increased to 32.6% in DET. In the current study, we have observed a direct association between the mother’s age and multiple pregnancy. Age is one of the risk factors for multiple pregnancy. The trend for this type of pregnancy exactly depended on the pattern of change in women's age at marriage (27, 28).

Adashi et al. (29), in a research conducted in the United States, found that 20% of the increase in twin births was attributed to the reproductive age of women. From the remaining 80%, ovulation with fertility drugs comprised 40 and 40% was attributed to IVF. In Denmark, an increase in multiple births was seen exclusively in women 30 years of age and older; most of the pregnancies were dizygotic. Blondel and Kaminski (30) have reported a one-quarter to one-third increase in twin pregnancies attributable to an increase in reproductive age in women. The increase in reproductive age is effective in twin birth of dizygotic, but the rate of monozygotic pregnancies is constant with changes in maternal age. This is due to the increase in gonadotropin levels with increasing age (31). Maximum follicle stimulation occurs between the ages of 35-39 years, after which ovarian function declines. Another finding of our study was the strong association between multiple pregnancy and a positive history of multiple births in the mother’s family. This association was not seen between multiple pregnancy and a positive history of multiple birth in the father’s family. In a casecontrol study in Italy the OR in women who had a history of multiple pregnancy in their first-degree relatives were 2.4 for dizygotes and 2.7 for monozygotes (14). Baldwin (32), observed these findings only in dizygotic twins. In a systematic review by Bortolus et al. (20) on factors that affected multiple births, an increase existed in the risk of multiple pregnancy in those with a history of multiple pregnancy in their first-degree relatives. The findings were confirmed for dizygotic twins. The mechanism of this association was explained in 1970 by Bulmer (33).

Our study was the first survey conducted with the large sample sizes from both public and private hospitals that had no selection bias (response proportion: 100%). We attempted to hold the same training session for interviewers (midwives) to minimize information bias. A pilot study carried out at the beginning of the study during over one week detected operational problems, and examined reliability and validity of the questionnaire. Our study has several limitations. First, the crosssectional nature of the study did not allow for conclusions on causality due to the because of temporality between the exposure and outcome. Second, in this study we only assessed multiple pregnancy without considering the type of multiple pregnancy (i.e., monozygote or dizygote).

## Conclusion

Based on the our study results, frequency of multiple pregnancy in older women, women with history of ART, and a history of multiple pregnancy in the mother’s family had a significant relationship with increased frequency of multiple pregnancy. We observed no significant relationship between the frequency of multiple pregnancy and other included variables.
